# Fœtal Malformation

**Published:** 1856-05

**Authors:** W. W. Adams

**Affiliations:** Clinton, Illinois


ARTICLE IV.—*Fœtal Malformation.* By W. W. Adams, M.D., of Clinton, Illinois.

The following case of malformation of a fœtus may not be uninteresting to the readers of the *Journal.*

On the evening of the 22d inst., at 10 o'clock, I was called to visit Mrs. M., in her eighth labor. She is a lady about medium size, spare, and nervo-sanguine temperament. Upon inquiry, I found her health had been on the decline for the past three or four months, during which time she had suffered frequent attacks of nervous headache, with occasional chills, and a general feeling of weakness and debility. I examined the abdomen, and found it very much distended, hard and unyielding. No motion had been felt that indicated to her a living child. The pains came on at regular intervals, of ten or fifteen minutes, but the uterus was so much upon the stretch, that it seemed to have lost its power to act upon the fœtus. An examination per vaginum, satisfied me that the case was likely to prove an interesting one. A consultation was determined, and Dr. C. Goodbrake was called. After deliberation, Dr. G. ruptured the membranes; the water gushed forth in a torrent, wetting the Dr. to his shoulder, and lying in a pool around the ladies person, until it run through the bed and down on the floor in a considerable quantity. The pains now ceased almost entirely. The breech could be distinctly felt in the upper strait. There was no hemorrhage. We allowed the patient to rest for half an hour. Two ounces of a strong infusion of ergot was then administered, which produced slight contractions. The dose was repeated in fifteen minutes, and in a short time the contractions of the uterus were sufficient to expel the fœtus, with another enormous discharge of liquor amnii.

The child was not dead, as we supposed, but it survived only a short time, and was a very singular case of malformation. Both hands were placed upon the styloid process, at right angles with the radias; the styloid process of the ulna and pisiform bone forming a very neat shaped heel; the thumb, scaphoid and trapezium bones were entirely wanting; the metacarpal bone of the forefinger articulated with the styloid process of the radius. The lower extremities were so dreadfully deformed that it would take much time and space to describe the deformity. The extremities were little else than skin and bone; the abdomen was very large, and filled with water; the umbilicus was surrounded by a dark livid circle, of about two inches in diameter, which was raised at least half an inch; the anus had the same peculiar appearance; the sphincter ani absent; the funis was about an inch in diameter, and distended with a clear transparent fluid, and when cut yielded but a few drops of dark blood; the edges of the ears were rolled forward, and had a crisped appearance, as if it had been done with a pair of heated tongs; the occipital bone was pressed under the parietal bone the entire length of the lamdoidal suture, leaving an offset of near one-eighth of an inch. I would consider this last a very interesting feature in the case. No doubt the pressure of the occipital bone upon the animal brain had been continuous for some time, and was the principal cause of the ascitic state of the body and smallness of the extremities.

My attention was first directed to this condition of the occiput by an article from the pen of J. M. Sims, M.D., of Montgomery, Alabama, under the head of Trismus Nascentium in young children. Since that time, I have seen and treated a number of cases of trismus, which, if recent, required but little treatment, and was relieved in a very few days by placing the child on its side upon a hard pillow. The bones soon gain their normal position, and the patient is relieved. But, if suffered to remain, a general wasting of the system takes place in defiance of every internal remedy, and death is the inevitable result.

Dr. Goodbrake and myself were called to visit a child not long since, between three and four months old, where we found the occiput in the same condition as in the above case. The parents informed us that it was at first a fine promising child, but soon became weakly and peevish, had frequent spasms; had taken a great deal of medicine, but continued to decline. When we saw it the extremities were very little larger than a man's finger. It died with spasms in about twelve hours. I would suggest to practitioners the propriety of examining the condition of the occiput when called to treat very young children; and where this condition prevails, ready relief will in most cases be obtained by placing the child on its side as above stated.

*Clinton, Ill., Feb.* 1856.

